# A Rare Case of Nodular Mantle Cell Lymphoma of the Gastrointestinal Tract Discovered During a Routine Colonoscopy With a Positive Response to R-CHOP Chemotherapy Regimen

**DOI:** 10.7759/cureus.42516

**Published:** 2023-07-26

**Authors:** Sukhjinder Chauhan, Jordan Valenta, Gundip S Dhillon, Preston Phan, Yongwoon Huh, Andre E Manov, Ann Wierman

**Affiliations:** 1 Internal Medicine, MountainView Hospital, Las Vegas, USA; 2 Medicine, Touro University Nevada, Henderson, USA; 3 Family Medicine, Valley Health System, Las Vegas, USA; 4 Internal Medicine, Sunrise Health Graduate Medical Education (GME) Consortium, Las Vegas, USA; 5 Hematology/Oncology, MountainView Hospital, Las Vegas, USA

**Keywords:** non-hodgkin lymphoma nhl, colonoscopy cecal, cecal lymphoma, treatment mantle cell lymphoma brief review, mantle cell lymphoma therapies, mantle cell lymphoma

## Abstract

This report describes the case of a 73-year-old female patient who presented with abdominal symptoms. A colonoscopy identified a cecal mass confirmed as mantle cell lymphoma (MCL). Imaging showed extensive lymph node involvement. The patient received rituximab, cyclophosphamide, hydroxydaunorubicin, vincristine, and prednisone (R-CHOP) chemotherapy, resulting in tumor reduction and adenopathy resolution. Despite a typically unfavorable prognosis associated with a high Ki-67 index, the patient responded well to chemotherapy and achieved a favorable outcome.

This case highlights the importance of early detection, appropriate treatment which in our case was R-CHOP, and personalized management approaches in addressing MCL.

## Introduction

Approximately 90% of malignant lymphomas are categorized as non-Hodgkin lymphomas (NHL), with the remaining 10% being classified as Hodgkin lymphoma (HL) [1]. NHL is considered the sixth most common type of cancer in the United States and the sixth leading cause of cancer deaths [2]. The primary causes of NHL remain unknown despite an increase in incidence, but established risk factors, including primary immunodeficiency disorders, human immunodeficiency virus (HIV), and organ transplantation, have been identified [3]. In developed countries, the most frequently diagnosed subtypes of NHL are diffuse large B-cell lymphoma (DLBCL) (about 30%) and follicular lymphoma (around 20%). Other subtypes occur less frequently, with less than 10% incidence [4]. Mantle cell lymphoma (MCL) comprises 3%-10% of adult NHL cases in Western countries, with a higher incidence among non-Hispanic whites in the US and higher in Caucasians than other ethnic groups [5-8].

In this report, we present the case of a 73-year-old Caucasian female who initially presented with symptoms of abdominal pain accompanied by vomiting, nausea, and unintentional weight loss. After further evaluation with colonoscopy, a cecal mass was discovered, and a biopsy result revealed a diagnosis of MCL. The patient was treated with rituximab, cyclophosphamide, hydroxydaunorubicin, vincristine, and prednisone (R-CHOP) and demonstrated a positive response to chemotherapy pending a hemicolectomy.

## Case presentation

**Figure 1:**
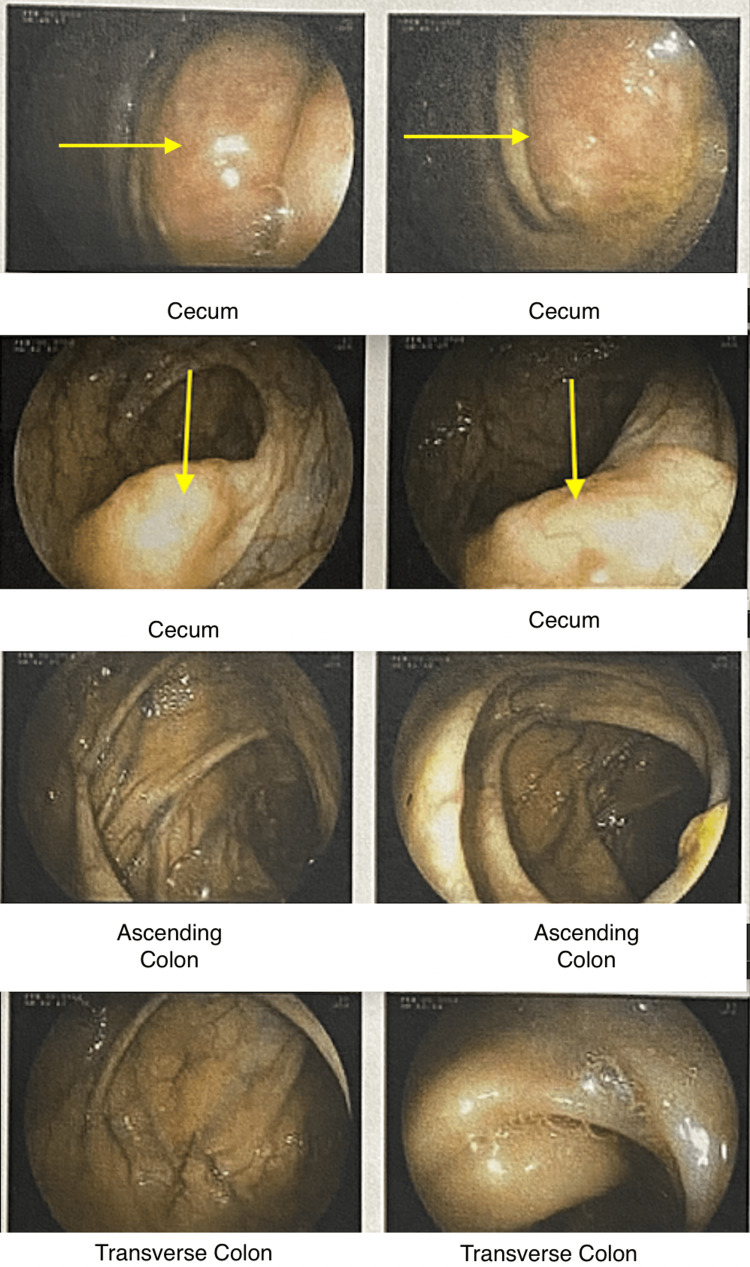
Colonoscopy reveals a cecal mass with a nodular appearance, marked with yellow arrows.

A 73-year-old Caucasian female with a past medical history of hypertension, hyperlipidemia, and obstructive sleep apnea presented to the hematology/oncology clinic to follow up on the biopsy results of a cecal mass. The cecal mass was initially identified during an outpatient colonoscopy, which was prompted by the patient's ongoing unintentional weight loss, intermittent constipation, abdominal pain, vomiting, and nausea (Figure 1). There were no reported polyps during the unremarkable screening colonoscopy that was conducted five years prior.

The immunohistochemical (IHC) analysis of a cecal mass demonstrated positive expression for CD5, CD20, cyclin D1, B-cell lymphoma-1 (BCL-1), and B-cell lymphoma-2 (BCL-2). The Ki-67 index, a commonly utilized marker for cell proliferation, was found to be between 15%-35%. Based on the IHC analysis, a diagnosis of MCL was confirmed. A bone marrow aspiration performed on the right iliac crest revealed that 1% of the cells were blasts or blastoid, 7% were promyelocytes, 19% were myelocytes/metamyelocytes, 35% were erythroid precursors, 11% were lymphoid cells, 1% were plasma cells, 16% were bands/segmented neutrophils, 10% were eosinophils, and less than 1% were monocytes and basophils. The myeloid to erythroid cell ratio was 1:5.1, which falls within the normal range of 1:1 to 2:1.

**Figure 2:**
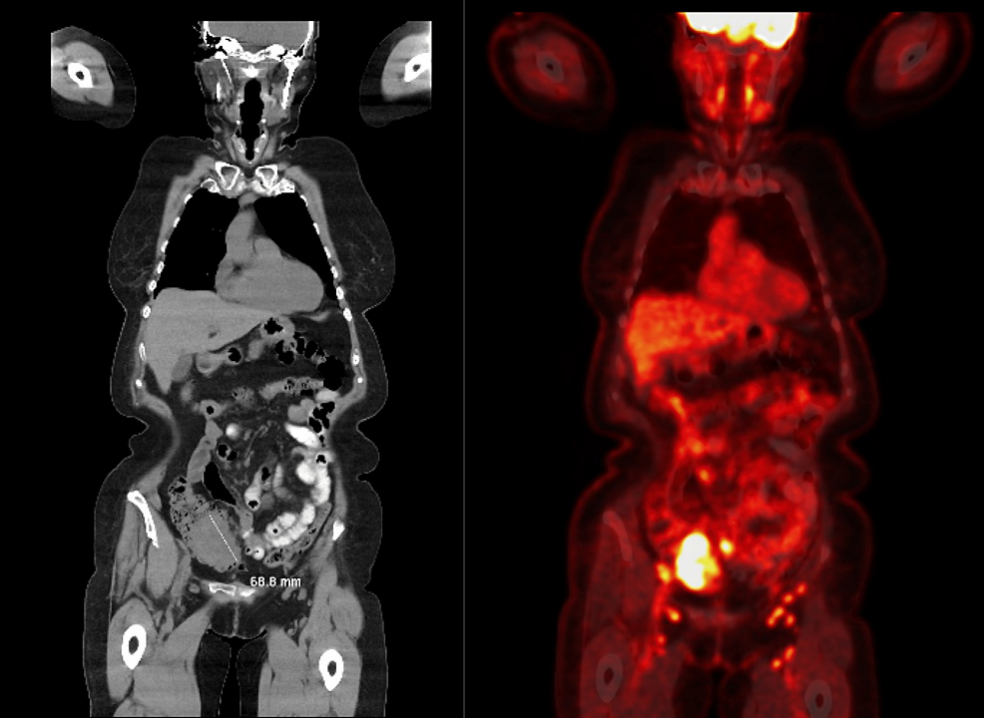
A coronal view CT scan displayed a cecal mass measuring approximately 7 cm with hyper-metabolic activity (left panel); positron emission tomography (PET) scans revealed extensive hyper-metabolic adenopathy extending from the abdomen to the neck, including a 1.8 cm lymph node in the right lower quadrant mesenteric region and peri-pancreatic area (right panel).

To further evaluate the extent of the disease, whole-body CT scans and positron emission tomography (PET) scans were obtained. The CT scan, displayed in Figure 2, demonstrated a cecal mass measuring up to approximately 7 cm (68.8 mm) on the left panel, and hypermetabolic activity on the PET scan on the right panel. The PET scans also revealed extensive hypermetabolic adenopathy, extending from the abdomen to the neck, with the largest affected lymph node in the right lower quadrant mesenteric region measuring 1.8 centimeters, as well as around the peripancreatic region (Figure 2).

**Figure 3:**
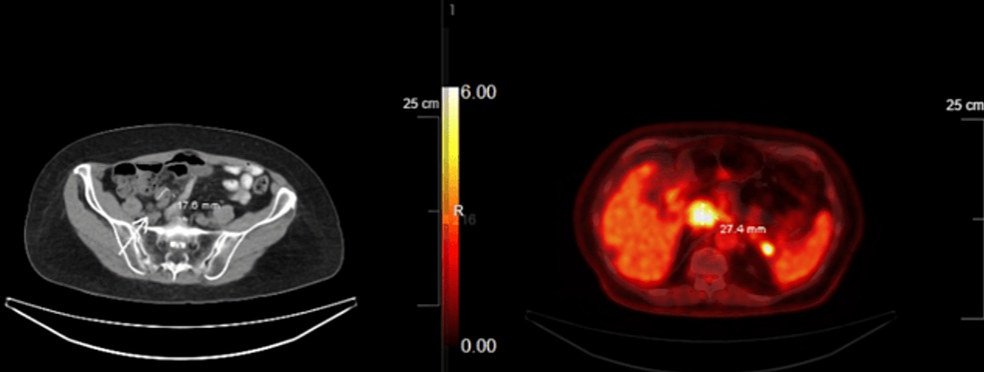
Additional CT sagittal views revealed a cecal mass with fluorodeoxyglucose-18 (FDG-18) uptake.

Further CT and PET images in sagittal views are shown in Figure 3.

**Figure 4:**
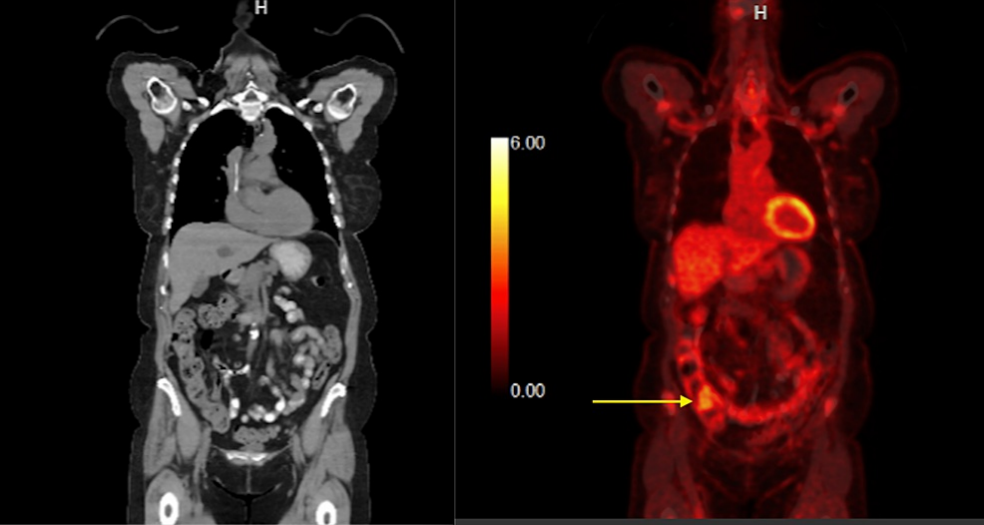
CT and PET scans reveal a significant reduction in size and minimal uptake on PET scan by cecal mass compared to pre-chemotherapy, indicating a positive response to chemotherapy (R-CHOP). PET: positron emission tomography; R-CHOP: rituximab, cyclophosphamide, hydroxydaunorubicin, vincristine, and prednisone.

The patient was informed of the findings and MCL diagnosis. The patient wanted to explore all further treatment options including chemotherapy. A plan was made to reduce the size of the tumor initially with induction therapy followed by a potential hemicolectomy. The induction chemotherapy regimen consisted of R-CHOP. The CT and PET scans were obtained three months after the chemotherapy to evaluate the response to chemotherapy. CT demonstrated a large cecal mass that had significantly decreased in size and exhibited minimal activity on the PET scan shown in Figure 4, as compared to Figures 2-3 prior to chemotherapy. This was indicative of a positive response to chemotherapy. The diffuse hypermetabolic adenopathy at the neck, chest, abdomen, and pelvis had also essentially resolved as previously seen in Figures 2-3.

**Figure 5:**
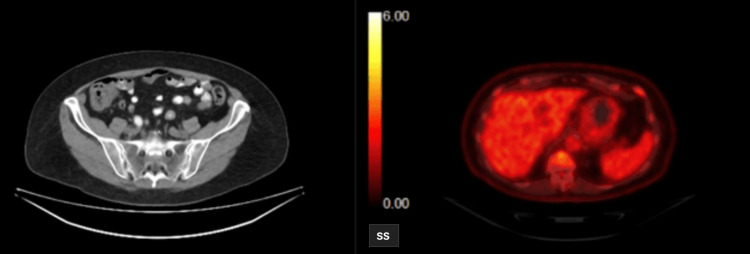
After six cycles of chemotherapy, CT and PET scans demonstrated no FDG-18 avid malignancy, including no presence of cecal mass. FDG-18: fluorodeoxyglucose-18; PET: positron emission tomography.

**Figure 6:**
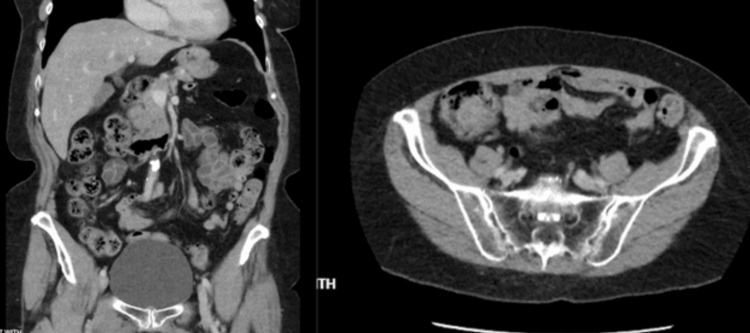
CT scans three months after completion of chemotherapy demonstrated no avid malignancy, including no presence of cecal mass.

The patient completed a total of six cycles of chemotherapy without any significant adverse effects. The CT and PET scan (shown in Figures 5-6) obtained after six cycles of chemotherapy demonstrated no fluorodeoxyglucose-18 (FDG-18) avid malignancy; specifically, no FDG-18 avid cecal mass was noted.

The patient was reassured of the new negative findings attributed to good chemotherapy response. The patient was referred to surgery for further follow-up for hemicolectomy. 

## Discussion

MCL is a subtype of adult NHL that accounts for 3%-10% of cases in Western countries, with a higher incidence among non-Hispanic whites in the US and a higher prevalence among Caucasians compared to other ethnic groups [5-8]. In the early 1980s, Weisenburger et al. and Palutke et al. made substantial contributions to the field by characterizing follicular lymphoma through their studies. They described a distinct phenotype of this disease, defined by the proliferation of atypical small lymphoid cells in a mantle-like pattern surrounding benign germinal centers. To identify this distinctive feature, Weisenburger et al. proposed the term "mantle zone lymphoma" and suggested that it constitutes the follicular counterpart of diffuse intermediate lymphocytic lymphoma [9-11].

A retrospective analysis of 80 cases of MCL was performed at the British Columbia Cancer Agency from 1988 to 1995. The study revealed that the average age of presentation for the patients was 64 years, with a median age of 65 years and a 2:3.1 male-to-female ratio; 76% of the patients were found to have extranodal involvement, with the most frequently impacted sites being the bone marrow (63%), peripheral blood (34%), gastrointestinal tract (10%), Waldeyer's ring (10%), and liver (8%). In 25% of cases, extranodal localization was the main presentation. Approximately 24% of the patients experienced B symptoms such as fever, night sweats, and weight loss, while 27% had elevated levels of lactate dehydrogenase (LDH) and 43% had normal LDH levels [12].

In most cases, MCL expresses B cell antigens such as CD19, CD20, CD5, and FMC7. It is a mature B cell neoplasia with aggressive behavior. Patients might present with nodal and extranodal disease and the disease is usually widely disseminated when diagnosed. Gastrointestinal involvement, bone marrow, and peripheral blood involvement are described. However, rare cases of MCL lacking the expression of CD5 or CD23 have been reported [13-15]. The molecular characteristics of MCL are marked by the overproduction of the protein cyclinD1 and the disturbance of several signaling pathways [16]. The t(11;14) (q13;q32) translocation is considered the origin of MCL, which leads to an overproduction of cyclin D1, a cell-cycle gene regulator. This protein works in conjunction with CDK4 and CDK6 to control the progression of the cell cycle and facilitate the transition from G1 to the S phase. The activated cyclin D1/CDK4 complex and cyclin E/CDK2 phosphorylate the retinoblastoma protein and release E2F to activate cell proliferation genes. However, the constitutive overproduction of cyclin D1 can result in excessive cell growth and cancer development [17,18]. It is important to note that the overexpression of cyclin D1 is not the only factor responsible for MCL [19]. Other genetic anomalies play a crucial role in MCL cells' early growth, spread, and resistance [20,21]. Mutations in the TP53 gene, prevalent in MCL and other forms of cancer, have been linked to adverse patient outcomes such as reduced survival and treatment response [22,23].

The morphological features of classical MCL, which account for 87% of the MCL, tend to consist of small to medium-sized lymphoid cells with unevenly shaped nuclei, limited cytoplasm, and barely noticeable nucleoli [24-27]. MCL can be differentiated from other types of lymphomas, such as follicular lymphoma, lymphoplasmacytic lymphoma, and chronic lymphocytic leukemia/small lymphocytic lymphoma, by the absence of cell types such as centroblasts, immunoblasts, prolymphocytes, and para-immunoblasts [24-27]. MCL growth pattern in the lymph nodes, spleen, or Waldayer's ring is commonly mantle-like, nodular, or widespread. In the early stages of the disease, the tumor invades the mantle zone followed by the invasion of the reactive germinal center and the formation of nodular patterns. These growth patterns have been observed through multiple biopsies and are thought to reflect the progression of MCL [24-26].

The variant forms of MCL include blastoid, pleomorphic, small cell, and marginal-like, with blastoid representing approximately 10%-15% of cases [27,28]. The blastoid form of MCL differs from classic MCL by its dispersed chromatin and cells that appear like lymphoblasts, with a cell division rate of at least 20-30 per 10 high-power fields. On the other hand, the pleomorphic variant of MCL features cells with various shapes (pleomorphic), including larger cells that exhibit oval to irregular nuclei, pale cytoplasm, noticeable nucleoli, and a high frequency of cell division, giving it a similar appearance to large cell lymphoma [29]. The modified Ann Arbor staging system is used to assess MCL. Stage I involves a single lymph node region or a localized extranodal site. Stage II involves two or more lymph node regions or localized extranodal sites on the same side of the diaphragm. Stage III involves lymph node regions or localized extranodal sites on both sides of the diaphragm. And finally, Stage IV is defined as diffuse or disseminated involvement of one or more extra lymphatic organs, with or without associated lymph node involvement or involvement of the liver or bone marrow [27].

The prognosis of a disease is determined by several factors, including its advanced stage, patient age, overall health, anemia, splenomegaly, high levels of β2-microglobulin and LDH, blastoid cytology, extranodal presentation, and presence of constitutional symptoms. The MCL International Prognostic Index (MIPI), a prognostic score mentioned in multiple studies, considers four key factors: age, performance status, LDH, and leukocyte count [30]. However, the most crucial prognostic indicator, independent of clinical features, is the rate of cell proliferation, measured by the Ki-67 index above 20% [30,31]. The Ki-67 proliferation index is the standard diagnostic tool in determining prognosis, with a value less than ten corresponding to a median survival of 42 months, 11%-40% corresponding to 30 months, and over 40% corresponding to 15 months [27, 31-33]. Radiotherapy has proven to be a successful single treatment option for early-stage MCL that is localized. This type of treatment has the potential to result in long-lasting remissions. On the other hand, the majority of patients with advanced-stage MCL need chemotherapy [27,34].

Rituximab, a monoclonal antibody can be added to the chemotherapy with or without a hematopoietic stem cell transplant (HSCT). The disease often relapses besides the initial response. The monoclonal antibody is less effective in MCL than in other B-cell lymphomas like follicular lymphoma and DLBCL. But combining rituximab with a chemotherapy regimen such as R-CHOP has shown to improve response rates, including complete response rates. The combined response rate to R-CHOP was 96% and 94%, respectively, with a median progression-free survival of 17-20 months, higher than the 75% response rate to CHOP alone without rituximab, with a median survival of 19 months [35,36]. The systematic review and meta-analysis of rituximab and chemotherapy versus chemotherapy alone showed that combination therapy might be superior [37]. Based on this evidence, rituximab should be considered for MCL patients undergoing chemotherapy. In patients with two or more relapses of their disease, Bruton’s tyrosine kinase (BTK) inhibitor and pirtobrutinib are usually used. A subset of patients with indolent disease might not require treatment for years.

In the case of our patient, the diagnosis of MCL was established based on IHC analysis which demonstrated positive expression for CD5, CD20, cyclin D1, BCL-1, and BCL-2. The Ki-67 index was found to be between 15%-35% which is usually indicative of a poor prognosis [30,31]. However, our patient responded very well to a chemotherapy regimen consisting of R-CHOP. After the successful completion of six cycles of chemotherapy, the patient's cecal mass was found to be completely resolved, and a significant resolution was observed in the mesenteric adenopathy.

## Conclusions

MCL is characterized by both nodal and extranodal involvement, and its prognosis is influenced by various factors, including disease stage and the Ki-67 proliferation index. Our patient's response to R-CHOP treatment demonstrated promising results, highlighting the effectiveness of this chemotherapy regimen. 

In conclusion, our case report highlights the positive response observed with the use of R-CHOP chemotherapy in the treatment of MCL. This rare case report adds to the existing literature on the management of this rare disease. The reporting of additional cases, and continued research and active participation in ongoing clinical trials will further enhance our understanding and refine the management strategies for MCL within the medical community. These collaborative efforts are essential for advancing our knowledge and improving patient care in this challenging condition.
